# Bioprospecting Sponge-Associated Marine Cyanobacteria to Produce Bioactive Compounds

**DOI:** 10.3390/toxins12020073

**Published:** 2020-01-23

**Authors:** Despoina Konstantinou, Eleni Mavrogonatou, Sevasti-Kiriaki Zervou, Panagiotis Giannogonas, Spyros Gkelis

**Affiliations:** 1 Department of Botany, School of Biology, Aristotle University of Thessaloniki, GR-541 24 Thessaloniki, Greece; kidespoi@bio.auth.gr (D.K.); elmavro@bio.demokritos.gr (E.M.); s.zervou@inn.demokritos.gr (S.-K.Z.); pgiannogonas@hotmail.com (P.G.); 2 Laboratory of Cell Proliferation and Ageing, Institute of Biosciences & Applications, National Centre for Scientific Research “Demokritos”, Patriarchou Grigoriou & Neapoleos, Agia Paraskevi, 15341 Athens, Greece; 3 Laboratory of Photo-Catalytic Processes and Environmental Chemistry, Institute of Nanoscience & Nanotechnology, National Centre for Scientific Research “Demokritos”, Patriarchou Grigoriou & Neapoleos, Agia Paraskevi, 15341 Athens, Greece

**Keywords:** NRPSs, PKSs, antibacterial, human cancer cell lines, cytotoxicity, *Leptothoe*

## Abstract

Marine cyanobacteria are considered a prolific source of bioactive natural products with a range of biotechnological and pharmacological applications. However, data on the production of natural compounds from sponge-associated cyanobacteria are scarce. This study aimed to assess the potential of sponge-associated cyanobacteria strains representing different taxonomic groups for the production of bioactive compounds and the biological activity of their extracts. Phylogenetic analysis of sponge-associated cyanobacteria and screening for the presence of genes encoding non-ribosomal peptide synthetases (NRPSs) and polyketide synthases (PKSs) were performed. Methanol extracts of the sponge-associated strains were analyzed for cyanotoxin production and tested for antioxidant activity and cytotoxic activity against several human cancer cell lines and pathogenic bacteria. PKS were detected in all sponge-associated strains examined, indicating the metabolic potential of the isolates. PKS genes were more ubiquitous than NRPS genes. Cyanotoxins (i.e., cylindrospermopsin, anatoxin-a, nodularin, and microcystins) were not detected in any of the sponge-associated cyanobacterial strains. Strains belonging to *Leptothoe*, *Pseudanabaena*, and *Synechococcus* were found to have activity mainly against *Staphylococcus aureus*. In addition, sponge-associated *Leptothoe* strains (TAU-MAC 0915, 1015, 1115, and 1215) were found to be highly cytotoxic and in most cases more effective against human cancer cell lines than against normal cells. Extracts with the most promising bioactivity deserve further investigation in order to isolate and identify the bioactive molecule(s).

## 1. Introduction

Cyanobacteria are photosynthetic prokaryotes with long evolutionary history, which is reflected in their worldwide distribution to a wide range of habitats including the extreme ones and in their high morphological, physiological, and metabolic diversity. They have gathered considerable attention for the production of toxins, as they form toxic blooms in freshwater bodies around the world, posing health risks to humans and animals [1]. However, cyanobacteria can also produce secondary metabolites with therapeutic properties and have emerged as a promising resource for the discovery of new drugs with potential pharmacological applications [2,3].

Marine cyanobacteria, in particular, are significant sources of structurally diverse marine secondary metabolites and are also among the few bacteria groups known to be chemically rich in providing new classes of compounds [2,4,5]. In fact, in the last decade, a plethora of natural compounds has been isolated and identified from marine cyanobacteria (more than 400 new natural compounds), mainly from filamentous stains [2,4,6]. These compounds include linear and cyclic peptides, linear lipopeptides, depsipeptides, fatty acid amides, swinholides, glicomacrolides, and macrolactones and show a broad spectrum of activities, such as antibacterial, antifungal, antiviral, cytotoxic, and anticancer activities [3,4,7].

Among potential bioactivities, anticancer effects have mostly attracted scientific interest, as cancer is the major cause of mortality worldwide. Nearly 100 compounds deriving from marine cyanobacteria have been found to induce cytotoxicity in several cancer cell lines [6,8], and currently 17 compounds are in different phases of clinical trials for cancer treatment. Some examples of potent anticancer compounds isolated from marine cyanobacteria, with known cytotoxic mechanisms, are dolastatins, cryptophycins, apratoxins, and aurilides [8]. The most famous example is brentuximab vedotin, a synthetic analog of dolastatin 10, which targets CD30 and microtubules, and is used for the treatment of Hodgkin’s lymphoma after approval by the Federal Drug Agency [9].

Cyanobacteria, in the marine environment, have been reported to live in association with a remarkable variety of hosts (e.g., fungi, ascidians, corals, and protists) [10], while the association formed with sponges has significantly attracted research interest from a biotechnological perspective (among others) in the last decades. Sponges are considered a rich source of bioactive natural products with potential biotechnological applications, namely in the pharmacological field, contributing to nearly 30% (more than 4850 compounds) of all marine natural products discovered [11]. Sponges are renowned for establishing symbiotic relationships with complex communities of microorganisms [12], in which symbiotic microorganisms have often been proposed to be the producers of the bioactive compounds [13,14]. Cyanobacteria have been recognized as a major group of sponge-associated microbes contributing to the production of pharmacologically relevant secondary metabolites [13].

According to the most recent review, cyanobacterial diversity reported from sponges is still underestimated; their taxonomy below the phylum level has received little attention while only few cyanobacteria strains isolated from sponge species are presently known [15]. Our recent research on sponge-associated cyanobacteria in the North Aegean Sea [15] resulted in the isolation of 15 cyanobacteria strains [15,16] and showed novel cyanobacteria diversity. Indeed, the investigation of five *Leptolyngbya*-like sponge-associated isolates based on a polyphasic approach (evaluation of molecular, chemical, and morphological data) revealed a novel marine genus, *Leptothoe* gen. nov., in which three new species of sponge-associated cyanobacteria, i.e., *Le. sithoniana*, *Le. kymatousa*, and *Le. spongobia*, were described. In the present study, we aimed to assess: (i) the production of bioactive compounds by sponge-associated cyanobacteria isolates representing different taxonomic groups; (ii) the biological activity of their extracts; and (iii) if closely affiliated strains could possess the same bioactivity.

## 2. Results

### 2.1. Molecular Characterization of Sponge-Associated Cyanobacteria Strains 

**Figure 1:**
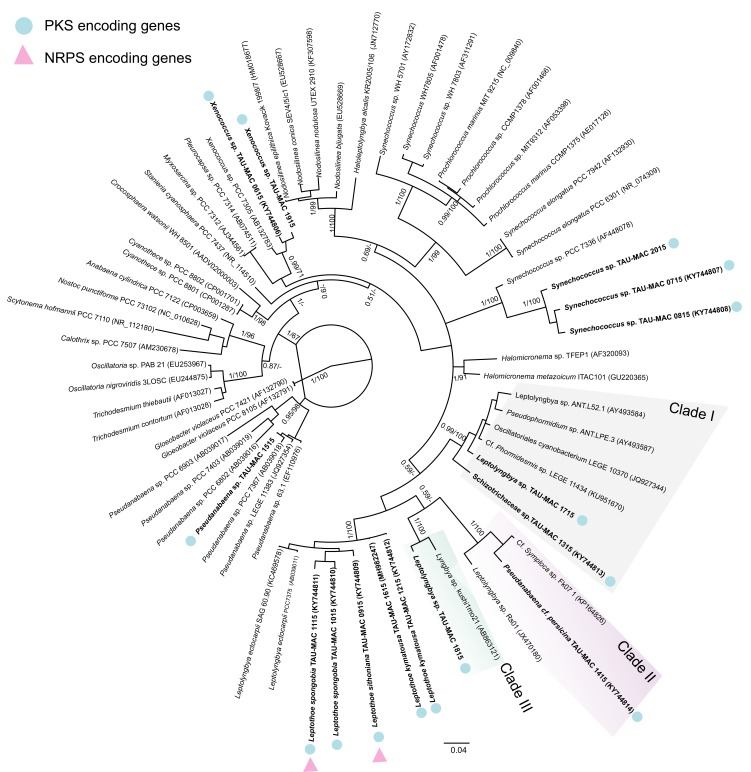
Phylogenetic relationships of the studied sponge-associated strains based on the 16S rRNA gene sequence (1056 bp), in relationship to representative strains of four cyanobacteria orders (Synechococcales, Pleurocapsales, Oscillatoriales and Nostocales), with *Gloeobacter violaceus* as outgroup. The tree was constructed with the Bayesian Inference (ΒΙ) method and the maximum-likelihood (ML) method; BI topology is demonstrated. Support values are indicated as posterior probability for Bayesian inference and bootstrap support for Maximum Likelihood analysis. Strains of the present study are indicated in bold, while GenBank accession numbers are indicated in brackets. Blue dots represent the detection of PKS encoding genes and pink triangles represent the detection of NRPS encoding genes. The bar represents 0.040 nucleotide substitutions per site.

Phylogenetic relationships of the 15 sponge-associated cyanobacteria strains (coccoid and filamentous) based on 16S rRNA gene sequence analysis showed that 13 strains belong to different lineages of the Synechococcales order and two strains to one lineage within Pleurocapsales (Figure 1). Two coccoid strains, namely TAU-MAC 0615 and 1915, assigned to *Xenococcus*, formed a distinct subclade, and clustered together with the reference strain of the same genus (*Xenococcus* sp. PCC 7305) within the Pleurocapsales order. Filamentous Synechococcales strains TAU-MAC 0915, 1015, 1115, 1215, and 1615 belonging to *Leptothoe* were affiliated to the strains Schizotrichaceae sp. TAU-MAC 1315, *Leptolyngbya* sp. TAU-MAC 1715, *Leptolyngbya* sp. TAU-MAC 1815, and *Pseudanabaena* cf. *persicina* 1415 strains were distributed in three distinct clades (Clades I–III) in the phylogenetic reconstruction shown in Figure 1. Inside Clades I–III, our strains clustered together with other strains of uncertain taxonomic status. The three sponge-associated *Synechococcus* strains (TAU-MAC 0715, 0815, and 2015) were placed in one subclade and together with the strain *Synechococcus* PCC 7376 were clustered in a well-supported clade (Figure 1). This clade was placed outside of other clades formed by marine *Synechococcus* and *Prochlorococcus*. Strain *Pseudanabaena* sp. TAU-MAC 1515 was placed in the same clade with other strains of the same genus of marine origin (Figure 1).

### 2.2. PCR Screening for PKS and NRPS Encoding Genes

To evaluate the potential of these cyanobacterial isolates to produce secondary metabolites, a PCR screening was performed for type I polyketide synthase (PKS) and non-ribosomal peptide synthetase (NRPS) genes. PKS encoding genes were detected in all sponge-associated cyanobacteria strains, while NRPS encoding genes were detected only in two strains of the species *Leptothoe spongobia* (TAU-MAC 1115) and *Le. sithoniana* (TAU-MAC 0915) (Figure 1).

### 2.3. Cyanotoxins

LC-MS/MS analysis results indicate that none of the targeted cyanotoxins, i.e., cylindrospermopsin (CYN), anatoxin-a (ATX-a), nodularin (NOD), microcystins (MCs), [D-Asp^3^]MC-RR, MC-RR, MC-YR, MC-HtyR, [D-Asp^3^]MC-LR, MC-LR, MC-HilR, MC-WR, MC-LA, MC-LY, MC-LW, and MC-LF, were present in detectable concentrations in the examined material of cyanobacterial strains associated with sponges. 

### 2.4. Antimicrobial Activity

Five sponge-associated cyanobacteria strains belonging to *Leptothoe*, *Pseudanabaena*, and *Synechococcus* genera inhibited the growth of three out of four heterotrophic bacteria tested (i.e., *Escherichia coli* 8879, *Pseudomonas aeruginosa* 12469, and *Staphylococcus aureus* 9518) (Table 1). The largest inhibition zone (10 mm) was observed in the cyanobacterial extract of *Pseudanabaena* cf. *persicina* TAU-MAC 1415 against *S. aureus* 9518. Inhibition zones in the rest of the heterotrophic bacteria were 5–8 mm.

### 2.5. Effects on Cell Lines

**Figure 2:**
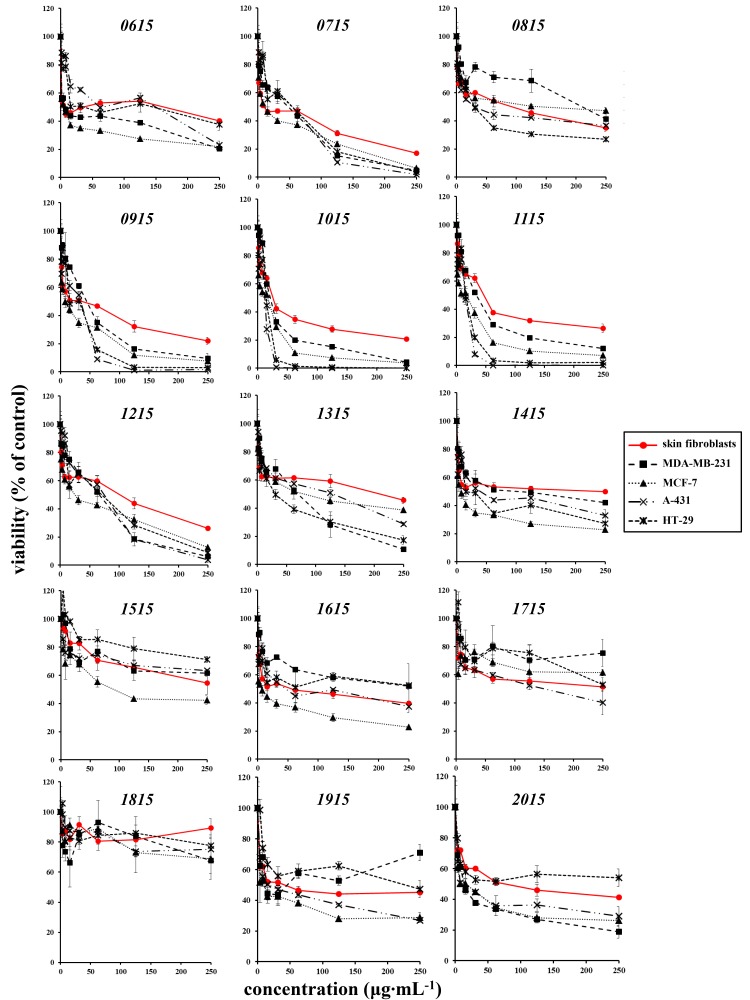
Effect of sponge-associated cyanobacterial extracts (TAU-MAC Culture Collection) on normal human skin fibroblasts’ and human cancer cells’ viability, as estimated by the MTT assay. Cells were plated in 96-well plates before the addition of each extract or DMSO alone at concentrations from 0 to 250 μg·mL^−1^ for 72 h. Optical density was measured at 550 nm and cell viability was calculated as a percent ratio of the samples solely treated with the respective DMSO concentration. Data presented are mean values ± standard deviations. At least two independent experiments were conducted in triplicates with similar results. One representative experiment is depicted here.

As shown in Figure 2, the majority of extracts were either non cytotoxic (i.e., extract TAU-MAC 1815), cytotoxic only at very high concentrations [i.e., extracts TAU-MAC 1515 and 1715 with a half maximal inhibitory concentration (IC_50_) that exceeded 100 or even 200 μg·mL^−1^], or non-selectively cytotoxic for cancer cells (i.e., extracts TAU-MAC 0615, 0715, 0815, 1315, 1415, 1615, 1915, and 2015). Four extracts however were found to be highly cytotoxic (i.e., extracts TAU-MAC 0915, 1015, 1115, and 1215). Among these, extracts TAU-MAC 1015 and 1115 were the most potent and selective for cancer cell lines, as presented in Table 2. Interestingly, all strains that showed high cytotoxicity belong to the genus *Leptothoe.*

**Figure 3:**
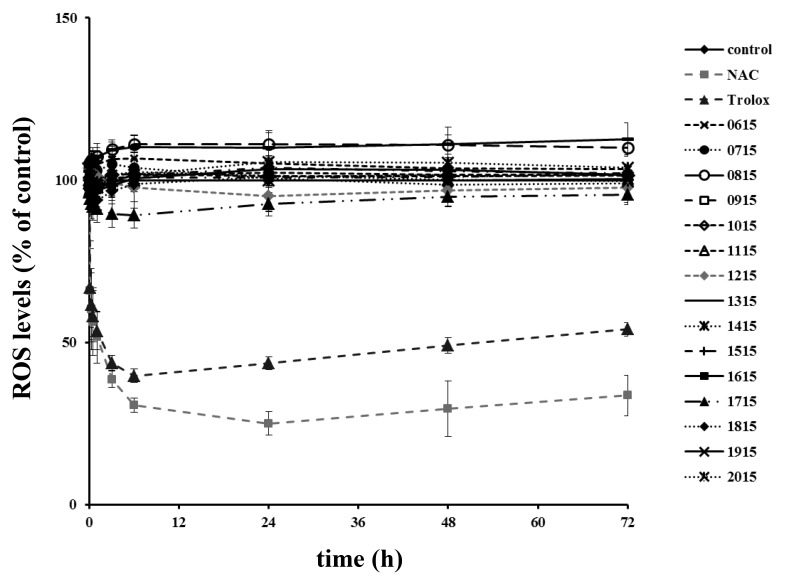
Estimation of intracellular ROS levels in normal human skin fibroblasts treated with the studied sponge-associated cyanobacterial extracts. Cells were plated in 96-well plates until they reached confluence. After a 24-h serum deprivation, cells were pre-incubated with 10 μΜ DCFH-DA and then exposed to the extracts at the following concentrations: 1015 and 1115 at 7.5 μg·mL^−1^; 0615, 0715, 0915, 1215, and 1315 at 15 μg·mL^−1^; 0815 and 1615 at 30 μg·mL^−1^; 1415, 1915, and 2015 at 50 μg·mL^−1^; 1515 and 1715 at 100 μg·mL^−1^; and 1815 at 200 μg·mL^−1^ or to the known antioxidants NAC (2 mM) and Trolox (20 μΜ). Fluorescence intensity was measured at the designated time points up to 72 h. Intracellular ROS levels were estimated as a percent ratio of the control. Results are expressed as mean values ± standard deviations.

For the assessment of the antioxidant potential, each extract was used at the highest concentration that—according to the MTT assay—had no severe impact on the viability of normal human skin fibroblasts. In contrast to the known antioxidants NAC and Trolox that were able to reduce intracellular reactive oxygen species (ROS) levels early on, no extract tested had an early or a late antioxidant effect on skin fibroblasts (Figure 3).

## 3. Discussion

Phylogenetic analysis performed in the present study corroborates our previous research [15]; the diversity of our sponge-associated isolates could be characterized as quite unique and their taxonomic status requires further investigation. The majority of the strains belong to distinct and well supported clades of Synechococcales (13/15) and Pleurocapsales (2/15), while in related studies sponge-associated cyanobacteria isolates have been found to belong exclusively to Synechococcales [17,18]. The *Leptolyngbya*-like strains TAU-MAC 1715 and 1815 as well as the strains Schizotrichaceae sp. TAU-MAC 1315 and *Pseudanabaena* cf. *persicina* [15] showed less than 94% similarity to the 16S rRNA gene sequences with the related filamentous genera *Leptothoe* [16], *Nodosilinea* [19], and *Haloleptolyngbya* [20]. The polyphyletic status of genus *Leptolyngbya* is well established, and, as has been underlined, there is still undiscovered diversity in *Leptolyngbya*-like cyanobacteria [16,19,20].

Our phylogenetic analysis was motivated by the aim of testing if closely affiliated strains could possess the same bioactivity. Interestingly, all cyanobacterial extracts (TAU-MAC 0915, 1015, 1115, and 1215) that were more cytotoxic belong to the newly described *Leptothoe* genus and to the three novel sponge-associated species *Le. sithoniana*, *Le. spongobia* and *Le. kymatousa* [16]. Previously, the strain *Phormidium ectocarpi* SAG 60.90 that also falls into the *Leptothoe* clade (Figure 1) has been found to produce hierridin B [21], a compound that was recently shown to have cytotoxic effects on human cancer cell lines [22]. The PKS clusters in all *Leptothoe* strains (TAU-MAC 0915, 1015, 1115, 1215, and 1615) of this study have also been previously detected in the other two strains belonging to the *Leptothoe* clade, *Phormidium ectocarpi* SAG 60.90 and *Leptolyngbya ectocarpi* PCC 7375 [23,24]. NRPS encoding genes were detected only in *Le. sithoniana* TAU-MAC 0915 and *Le. spongobia* TAU-MAC 1115, as PKS genes seem to be more ubiquitous than NRPS genes in marine cyanobacteria [25]. It should be noted that *Le. sithoniana* TAU-MAC 0915 and *Le. spongobia* TAU-MAC 1115 are the only strains containing both NRPS and PKS genes and showing both antibacterial and cytotoxic activities. By contrast, the rest of sponge-associated strains that showed either cytotoxic or antibacterial activity contained only PKS genes. PKS and NRPS gene clusters have been linked with the production of bioactive molecules [2,5] and, further, advances in bioinformatics have revealed that there are still cryptic biosynthetic gene clusters related to the production of novel natural products [5]. It is interesting that two strains belonging to the species *Le. spongobia* have differences since one has NRPS genes (TAU-MAC 1115) and the other (TAU-MAC 1015) does not. This is not surprising as infraspecific and/or intra-strain variation in the content of biosynthetic genes as well as in the production of secondary metabolites is well documented in cyanobacteria [1]. Previous studies detecting the presence of PKS and NRPS encoding genes in cyanobacteria reported more often the presence of these genes in filamentous than coccoid strains [24,25,26], but no difference was observed in our sponge-associated strains, at least for the PKS cluster; both coccoid and filamentous strains were found to possess these genes. It is worth mentioning that our coccoid strains (*Synechococcus* TAU-MAC 0715, 0815, and 2015 and *Xenococcus* TAU-MAC 0615 and 1915) were grouped into distinct clades with strains of the Pasteur Culture Collection (*Synechococcus* PCC 7336 and *Xenococcus* PCC 7305, respectively), in which the presence of PKS and NRPS encoding genes has been previously detected [26]. A great fraction of the biosynthetic gene clusters (NRPS and PKS) in cyanobacteria and other prokaryotes remains unknown, similar to other complex marine organisms such as dinoflagellates [27].

Regarding cyanotoxin production, free-living cyanobacteria strains isolated from Greek freshwater ecosystems are well documented to produce mainly microcystins (e.g., [28,29]), while the presence of cyanotoxins (i.e., CYN, ANA, NOD, and MCs) was not detected in our sponge-associated cyanobacteria isolated from the Aegean Sea. However, in the strain *Leptothoe spongobia* TAU-MAC 1115, isolated in January 2015 and first analyzed for the synthesis of microcystin in June 2016, the presence of MC-RR (0.14 μg·g^−1^ dw) was identified by the LC-MS/MS analysis [16]. In a recent study, an example of a cyanobacterial strain which lost the ability to produce microcystin under laboratory conditions was shown [30]. Since the LC-MS/MS analytical method [31] used herein is able to unequivocally identify several cyanotoxins (i.e., CYN, ANA, NOD, and MCs), most of them known for their cytotoxic effects [32], the negative results obtained for the tested strains indicate that the observed bioactivities may be caused from other compounds (unknown or not identified).

The potential of sponge-associated cyanobacteria isolates to show antibacterial activities was evaluated for the first time herein, and strains belonging to *Leptothoe*, *Pseudanabaena*, and *Synechococcus* genera were mainly inhibitory for the Gram-positive bacterium *Staphylococcus aureus*. Antibacterial activities against *S. aureus* were also observed in a recent study of our group testing the extracts of freshwater cyanobacteria [28]. Antimicrobial activities of cyanobacteria have been mostly reported from marine filamentous strains (e.g., [33,34]), while Martins et al. [35] suggested that marine *Synechocystis* and *Synechococcus* genera are a source of antibiotic compounds. Swain et al. [36] described 121 compounds with antimicrobial activity among cyanobacteria, but few cyanobacterial compounds and derivatives have gone up into clinical trials and no cyanoderivative has been approved by Food and Drug Administration (FDA), yet. Bacteria become drug resistant, thus novel compounds from cyanobacteria could suitably be exploited in drug development. 

The high proportion of marine cyanobacterial compounds revealed as cytotoxic has fueled the research effort for screening more and more marine cyanobacteria strains aiming to reveal new cytotoxic compounds. To date, marine cyanobacteria that have shown strong cytotoxicity effects against human cancer cell lines belong to the following genera: *Lyngbya* and the recently separated *Moorea* and *Okeania* (previously identified as *Lyngbya*) [33,37,38], *Symploca* [39,40] and the recently separated *Caldora* (previously identified as *Symploca*) [41], *Geitlerinema* [42], *Phormidium* [43], *Oscillatoria* [44], *Leptolyngbya* [45], *Nodosilinea* [46], *Pseudanabaena* [46], *Cyanobium* [23], *Synechocystis* [46], and *Synechococcus* [46]. In the present study, we add another genus showing a cytotoxic effect against human cancer cell lines, the novel genus *Leptothoe* [16]. In fact, our sponge-associated *Leptothoe* strains (TAU-MAC 0915, 1015, 1115, and 1215) were found to be highly cytotoxic, and the newly described sponge-associated species *Le. spongobia* [16] seems to be the most promising since the extracts of both *Le. spongobia* strains (TAU-MAC 1015 and 1115) were the most potent. As it comes out, these sponge-associated strains seem to produce cytotoxic compounds and deserve further research. Some examples of potent anticancer compounds isolated from cyanobacteria, with known cytotoxicity mechanisms, are dolastatins, cryptophycins, apratoxins, and aurilides [2,8]. On the other hand, a significant number of anticancer compounds deriving from cyanobacteria with unknown mode of cytotoxic action exists, including dragonamides, minutissamides, and almiramides [8].

Thus far, the most recent approaches to explore the bioactivity of marine cyanobacteria have mainly addressed free-living forms, due to the limitation posed by the inability to isolate and culture symbiotic microorganisms. Therefore, few studies assessing the biotechnological potential of sponge-associated cyanobacteria exist. Previously, three *Leptolyngbya* and *Synechococcus* strains isolated from the Mediterranean sponge *Petrosia ficiformis* were investigated for their bioactive properties, based on bioassays with human erythrocytes, *Artemia salina* nauplii, and *Paracentrotus lividus* gametes and showed cytotoxic activity [18]. The same bioassays were performed in 12 recently isolated cyanobacteria from sponges of the Portuguese coast and eight extracts showed a promising potential [47]. Herein, sponge-associated cyanobacterial strains were tested for the first time against pathogenic bacteria species and human cancer cell lines, and some of them seem to be promising.

## 4. Conclusions

In the present study, we performed an assessment on the potential of sponge-associated cyanobacteria isolates, representing different taxa of Synechococcales and Pleurocapsales orders, to produce bioactive compounds and the biological activity of their extracts. PKS genes were detected in all sponge-associated strains examined, indicating the metabolic potential of the isolates, whereas they were more ubiquitous than NRPS genes. Sponge-associated cyanobacteria strains belonging to *Leptothoe*, *Pseudanabaen*a, and *Synechococcus* were found to have activity mainly against *Staphylococcus aureus*; *Pseudanabaena* cf. *persicina* TAU-MAC 1415 showed the strongest inhibitory effect. Moreover, four out of five sponge-associated *Leptothoe* strains were found to be cytotoxic. Taking into account previous works that screened strains corresponding to the *Leptothoe* clade for cytotoxicity and are discussed herein, we could hypothesize that this lineage is enriched with core gene clusters involved in the biosynthesis of cytotoxic compounds. Although these findings should be regarded as indicative, they highlight the metabolic potential of these sponge-associated cyanobacterial isolates to produce natural products and allow the selection of the most promising strains for further studies to isolate and identify the bioactive molecule(s).

## 5. Materials and Methods 

### 5.1. Cyanobacterial Isolates and Culture Conditions

Fifteen strains of sponge-associated cyanobacteria of the Thessaloniki Aristotle University Microalgae and Cyanobacteria (TAU-MAC) culture collection [48] were used in this study. Among these, ten sponge-associated cyanobacteria strains have been previously isolated from sponges and identified as members of the taxa *Synechococcus*, *Xenococcus*, *Pseudanabaena*, Schizotrichaceae, and the newly described genus *Leptothoe* [15,16], while five strains were characterized in the present study (Table 3). The cultures were grown in MN medium with the addition of inorganic nitrogen [49]. Cultures were maintained under white fluorescent light (20 μmol·photons·m^−2^·s^−1^) with a 12:12 h light: dark cycle at 20 ± 1.0 °C.

### 5.2. DNA Extraction, PCR Amplification and Sequencing

Cultured sponge-associated cyanobacteria cells (1.5 mL) were harvested during exponential growth phase by centrifugation. The cells were resuspended in 800 μL of lysis buffer [2% (*w*/*v*) Cetyl Trimethyl Ammonium Bromide (CTAB), 100 mM Tris⎯HCl, 1.4 M NaCl, 1% (*w*/*v*) polyvinylpyrrolidone (PVP), and 20 mM disodium salt of ethylenediaminetetraacetic acid] [50] and incubated at 65 °C for 1 h (gently shacked every 10 min). The sample of lysed cells was extracted with chloroform–isoamyl alcohol using the protocol described by Atashpaz et al. (2010) [50]. Partial 16S rRNA gene was amplified from strains TAU-MAC 1515, 1715, 1815, 1915, and 2015 using CYA106F and 23S30R primers [51,52], as described by Konstantinou et al. [15]. Amplification of the 16S rRNA gene was performed according to the methods described previously [15,16]. PCR amplifications of PKS and NRPS gene clusters from all 15 cyanobacteria strains were performed using the degenerate oligonucleotide primer pairs DKF/DKR [53] and MTF2/MTR [54], respectively. PCR reactions were carried out using an Eppendorf MasterCycler Pro (Eppendorf, Hamburg, Germany), and were prepared in a volume of 25 μL containing 5× PCR buffer, 200 μM MgCl_2_, 0.2 mM of each deoxynucleotide triphosphate, 0.5 μΜ of each of the primers, 0.8 U of taq DNA polymerase, and 30–80 ng of genomic DNA, determined with the Nanodrop 2000 (Thermo Fisher Scientific, Waltham, MA, USA). Initial denaturation at 94 °C for 4 min was followed by 30 cycles of denaturation at 94 °C for 30 s, annealing at 58 °C (DKF/DKR) or 48 °C (MTF2/MTR) for 30 s, extension at 72 °C for 1 min, and a final extension at 72 °C for 7 min. PCR products were separated on a 1.5% (*w*/*v*) agarose gel by electrophoresis, visualized with Μidory Green (Nippon Genetics Europe GmbH, Düren, Germany), and photographed under UV transillumination.

PCR products of the 16S rRNA gene were purified using the Nucleospin Gel and PCR clean up (Macherey-Nagel, Düren, Germany) kit. The purified products were sequenced using the same primer pairs as in the PCR amplifications with ABI 3730xl DNA Analyzer. Partial 16S rRNA sequence data were obtained from TAU-MAC 1515, 1715, 1815, 1915, and 2015 and processed with BioEdit (Ibis Biosciences 1997–2015) software. Chimera check was performed for sequences using Pintail [55]. Sequences were deposited in the GenBank database under the accession numbers MN833625–MN833629.

### 5.3. Phylogenetic Analysis

The 16S rRNA gene sequences of all 15 sponge-associated cyanobacteria strains and sequences that showed >97% sequence similarity via BLAST searches (at least 1065 nucleotides) were included in the analysis along with representative sequences of taxa of four major cyanobacteria orders (Synechococcales, Pleurocapsales, Oscillatoriales, and Nostocales). Two sequences of the unicellular *Gloeobacter violaceus* (GenBank acc. No. AF132790 and AF132790) were used as outgroup, because genus *Gloeobacter* is distantly related to all cyanobacteria taxa [56]. Multiple sequence alignments were performed in MEGA v. 7.0 [57] using ClustalW [58]. Phylogenetic trees were constructed using Maximum Likelihood (ML) and Bayesian inference (BI). The GTR+I+G model was determined in jModelTest 0.1.1 [59] as the most appropriate, based on both the Bayesian and Akaike information criterion, and was used for ML and BI analyses. The ML analysis was performed in MEGA v. 7.0 [57]. Bootstrap resampling was performed on 1000 replicates. Bayesian analysis was conducted using MrBayes 3.2.6 [60]. Four Metropolis-coupled MCMC chains (three heated chains and one cold) were run for 10,000,000 generations, the first 2,500,000 generations were discarded as burn-in, and the following datasets were sampled every 1000th generation. Phylogenetic trees were visualized using the FigTree (V1.4.3) software.

### 5.4. Cyanotoxin Analysis Using LC-MS/MS

Lyophilized cyanobacterial cells were extracted for the analysis of cylindrospermopsin (CYN), anatoxin-a (ATX-a), nodularin (NOD), and 12 microcystins (MCs), as described in detail by Christophoridis et al. [61]. Briefly, 10 mg dry weight (dw) of culture material were suspended in 1.5 mL 75% (*v*/*v*) aqueous methanol, sonicated for 15 min in a sonication bath, centrifuged for 10 min at 4000 rpm, and the supernatant was collected. The pellet was re-extracted twice, with 1.5 mL 75% (*v*/*v*) aqueous methanol and 1.5 mL n-butanol. Then, 1.5 mL of the pooled supernatants were evaporated to dryness under nitrogen stream. The residue was dissolved in 0.5 mL of 5% (*v*/*v*) aqueous methanol and sonicated for 5 min.

LC-MS/MS analysis of CYN, ATX-a, NOD, and 12 MCs ([D-Asp^3^]MC-RR, MC-RR, MC-YR, MC-HtyR, [D-Asp^3^]MC-LR, MC-LR, MC-HilR, MC-WR, MC-LA, MC-LY, MC-LW, and MC-LF) was performed on a Finnigan Surveyor LC system, equipped with a Finnigan Surveyor AS autosampler (Thermo Fisher Scientific), coupled with Finnigan TSQ Quantum Discovery Max triple-stage quadrupole mass spectrometer (Thermo Fisher Scientific), using positive electrospray ionization (ESI) in multiple reaction monitoring (MRM) mode. Xcalibur software 2.1 SP 1160 was used to control the system and for data acquisition. The determination of 15 cyanotoxins was carried out according to the LC-MS/MS method described by Zervou et al. [31]. Detection limits (LODs) of the applied method were 0.1 μg·g^−1^ dw for CYN, 0.3 μg·g^−1^ dw for ATX-a, 0.2 μg·g^−1^ dw for NOD, and varied from 0.1 to 0.7 μg·g^−1^ dw for MCs.

### 5.5. Extract Preparation for Assays

Sponge-associated cyanobacteria cells were harvested at the exponential growth phase (between 25 and 35 days of growth) by centrifugation and were freeze-dried. Extract preparation for antimicrobial assays was done as follows: lyophilized culture material (~30 mg dry weight) was dissolved in 8 mL of 75% (*v*/*v*) aqueous methanol and sonicated for 15 min. Further, the extraction procedure was performed according to the protocols described by Gkelis et al. [28,62].

Extract preparation for the cytotoxicity assay and for the estimation of the antioxidant potential was done as follows: lyophilized culture material (~50 mg dw) was extracted with 20 mL 90% (*v*/*v*) aqueous methanol. The mixture was vortexed for 3 min, sonicated for cell lysis in a sonication bath for 15 min, and vortexed again for 15 min. Supernatant was collected in a round-bottom flask, after centrifugation for 5 min at 4000 rpm. The pellet was further extracted with 20 mL methanol, vortexed for 15 min, and centrifuged for 5 min at 4000 rpm. Supernatants were pooled in the same round-bottom flask and concentrated under vacuum using a rotary evaporator (35–40 °C). Residue was transferred assisted by small portions of methanol in a pre-weighted vial and evaporated to dryness under nitrogen steam. Dry weight of final extract was measured in order to evaluate the bioactivity.

### 5.6. Agar Disc Diffusion Assay

Four heterotrophic bacteria strains, *Escherichia coli* 8879, *Pseudomonas aeruginosa* 12469, *Bacillus subtilis* 3610, and *Staphylococcus aureus* 9518, were used for antibacterial assays. Cultures were grown in Mueller Hinton Broth (MH, Merck, Kenilworth, NJ, USA). Sterilized Whatman^®^ filter paper discs (6 mm diameter) (Merck) were impregnated with the cyanobacterial extract and placed on MH II Agar (Merck) plates seeded with a lawn of the tested microorganism. Double-distilled water (ddH_2_O) was used as a negative control. After 48 h of incubation at 37 °C, the plates were analyzed for inhibition zones, which were measured for every cyanobacterial extract. 

### 5.7. Human Cell Lines and Cell Culture Conditions

Normal human skin fibroblasts (AG01523) used in this work were obtained from the Coriell Cell Repositories (Camden, NJ, USA). Human breast cancer epithelial cells MDA-MB-231 and MCF-7, human skin cancer epithelial cells A-431 and human colon cancer epithelial cells HT-29 were purchased from the American Type Culture Collection (ATCC, Rockville, MD, USA). Skin fibroblasts and cancer cells were routinely cultured in DMEM containing penicillin (100 U/mL), streptomycin (100 μg·mL^−1^), and 15% or 10% (*v*/*v*) fetal bovine serum (FBS), respectively (all from Gibco, Thermo Fisher Scientific). Cultures were maintained at 37 °C and 5% CO_2_ and cells were subcultured when necessary using a trypsin/citrate (0.25%/0.30%, *w*/*v*) solution.

### 5.8. Cytotoxicity Assay

Obtained extracts were dissolved in dimethyl sulfoxide (DMSO, Merck, Darmstadt, Germany) at a final concentration of 20 mg·mL^−1^ and their effect on cell viability was assessed using the MTT assay, as previously reported [63]. In brief, cells were plated in 96-well plates before the addition of two-fold serial dilutions of the extracts to achieve final concentrations ranging from 0 to 250 μg·mL^−1^. The same concentrations of DMSO alone were also tested to rule out the possibility of a cytotoxic effect caused by the solvent itself. Cells were incubated with DMSO or the extracts at 37 °C and 5% CO_2_ for 72 h. Optical density was measured at 550 nm and cell viability was presented as percent ratio of the samples not treated with the extracts. IC_50_ values were calculated for the four most potent extracts.

### 5.9. Estimation of the Antioxidant Potential

Extracts’ putative antioxidant potential was estimated with the 2′,7′-dichlorofluorescein-diacetate (DCFH-DA) assay, as reported previously [63,64,65] with slight modifications. In detail, human skin fibroblasts were plated in 96-well plates in DMEM supplemented with 15% (*v*/*v*) FBS and were grown until they reached confluence. Cells were then deprived of serum by medium change to DMEM containing 0.1% (*v*/*v*) FBS for 24 h, before the addition of 10 μM of DCFH-DA in each well for 1.5 h. Then, cells were incubated with selected non-cytotoxic concentrations of the extracts or DMSO alone, as well as with the known antioxidants N-acetyl-L-cysteine (NAC, Merck) and 6-hydroxy-2,5,7,8-tetramethylchromane-2-carboxylic acid (Trolox, Merck) that served as positive controls. Fluorescence intensity (excitation wavelength: 485 nm, emission wavelength: 520 nm) was measured by an Infinite 200 Tecan microtiter-plate photometer (Tecan Trading AG, Switzerland) and alterations in intracellular ROS levels were expressed as a percent ratio of the samples not treated with the extracts.

### 5.10. Statistical Analysis

Statistical analysis was performed with Statgraphics Centurion software (Manugistics Inc., Rockville, MD, USA). IC_50_ values of selected extracts were compared using the ANOVA test and Fisher’s LSD post hoc analysis (*p* < 0.05). Means not sharing common letters are significantly different from each other.

## Figures and Tables

**Table 1:** Effect of crude cyanobacterial extracts on the growth of bacteria. Inhibition zones were measured in mm diameter including the diameter of the paper disk (− no inhibitory effect; + <10 mm; ++ 10 mm).

Cyanobacteria Strain (TAU-MAC)	Bacteria			
	Gram+		Gram-	
	*Staphylococcus aureus* 9518	*Pseudomonas aeruginosa* 12469	*Escherichia coli* 8879	*Bacillus subtilis* 3610
*Xenococcus* sp. 0615	−	−	−	−
*Synechococcus* sp. 0715	−	−	−	−
*Synechococcus* sp. 0815	+	−	−	−
*Leptothoe sithoniana* 0915	+	−	−	−
*Leptothoe spongobia* 1015	−	−	−	−
*Leptothoe spongobia* 1115	+	−	−	−
*Leptothoe kymatousa* 1215	−	−	−	−
Schizotrichaceae sp. 1315	−	−	−	−
*Pseudanabaena* cf. *persicina* 1415	++	+	+	−
*Pseudanabaena* sp. 1515	−	−	−	−
*Leptothoe kymatousa* 1615	+	−	−	−
*Leptolyngbya* sp. 1715	−	−	−	−
*Leptolyngbya* sp. 1815	−	−	−	−
*Xenococcus* sp. 1915	−	−	−	−
*Synechococcus* sp. 2015	−	−	−	−

**Table 2:** IC_50_ values (means ± standard deviations in μg·mL^−1^) of sponge-associated cyanobacterial extracts TAU-MAC 0915, 1015, 1115, and 1215. Values not sharing a common letter among cell lines tested after ANOVA and Fisher’s LSD analysis (*p* < 0.05) are significantly different.

	IC_50_ (μg·mL^−1^)	*p* Value (ANOVA test)	Fisher’s LSD
**TAU-MAC 0915**			
**Skin fibroblasts**	28.76 ± 18.79	0.0149	*ab*
**MDA-MB-231**	44.58 ± 2.99		*b*
**MCF-7**	9.68 ± 0.58		*c*
**A-431**	29.12 ± 1.37		*ab*
**HT-29**	26.45 ± 8.39		*ac*
**TAU-MAC 1015**			
**Skin fibroblasts**	45.74 ± 18.40	0.0439	*a*
**MDA-MB-231**	26.17 ± 7.41		*b*
**MCF-7**	20.53 ± 9.68		*b*
**A-431**	14.03 ± 1.48		*b*
**HT-29**	17.56 ± 0.69		*b*
**TAU-MAC 1115**			
**Skin fibroblasts**	45.25 ± 3.76	0.0000	*a*
**MDA-MB-231**	37.26 ± 1.44		*b*
**MCF-7**	14.88 ±4.55		*c*
**A-431**	15.86 ± 0.73		*c*
**HT-29**	18.39 ± 1.37		*c*
**TAU-MAC 1215**			
**Skin fibroblasts**	112.58 ± 7.86	0.0102	*a*
**MDA-MB-231**	65.64 ± 2.43		*b*
**MCF-7**	34.95 ± 8.71		*b*
**A-431**	42.66 ± 31.55		*b*
**HT-29**	55.38 ± 17.51		*b*

**Table 3:** Cyanobacteria strains isolated from different sponge-hosts and habitat types from the North Aegean Sea, Greece (39.951° N, 23.685° E).

Strain (TAU-MAC)	Taxonomy	Sponge-Host	Habitat-Type	Reference
0615	*Xenococcus* sp.	*Ircinia variabilis*	overhang	[15]
0715	*Synechococcus* sp.	*Axinella cannabina*	overhang	[15]
0815	*Synechococcus* sp.	*Axinella damicornis*	overhang	[15]
0915	*Leptothoe sithoniana*	*Petrosia (Petrosia) ficiformis*	rocky reef	[16]
1015	*Leptothoe spongobia*	*Dysidea avara*	rocky reef	[16]
1115	*Leptothoe spongobia*	*Acanthella acuta*	overhang	[16]
1215	*Leptothoe kymatousa*	*Chondrilla nucula*	rocky reef	[16]
1315	Schizotricaceae sp.	*Aplysina aerophoba*	rocky reef	[15]
1415	*Pseudanabaena* cf. *persicina*	*Axinella damicornis*	overhang	[15]
1515	*Pseudanabaena* sp.	*Spirastrella cunctatrix*	rocky reef	This study
1615	*Leptothoe kymatousa*	*Chondrilla nucula*	rocky reef	[15]
1715	*Leptolyngbya* sp.	*Hexadella racovitzai*	overhang	This study
1815	*Leptolyngbya* sp.	*Agelas oroides*	rocky reef	This study
1915	*Xenococcus* sp.	*Haliclona (Halichoclona) fulva*	overhang	This study
2015	*Synechococcus* sp.	*Stryphnus ponderosus*	rocky reef	This study
